# 

**Published:** 1905-05

**Authors:** 


					﻿SOUVENIR EDITION.
Vol. XX. i
MAY, 1905.
No. 11.
.Ttexivs
n .	<	• f
Medical Journal.
os
o
z>
o
Oi
4-»'
03
4-*
1ft
4-*
tj
o
Z3
$3
X2
4->
§
o
1ft
w
£x
is
<p
o
o’
o
r-b
O
H
so
«>
>♦
?
Q
$3
a>
2
«♦
*“*?
N>
4-
■
N>
CO
§
o.
Subscription, $i.oo a Year, i/ Advance.
Single Copies, io CeNt$ CtyDlF'
PUBLISHED AT AU8TIN, TEXAS, BY
F. E. DANIEL, M. D.,
Proprietor.
PRINTED BY THE VON BOBCKMANN-JONE8 CO.
Entered at the Poetoffice at A usttn, Texas, as Second-class Mail Matter.
				

